# A Qualitative Approach to Understanding Real-World Electronic Cigarette Use: Implications for Measurement and Regulation

**DOI:** 10.5888/pcd13.150502

**Published:** 2016-01-14

**Authors:** Maria Cooper, Melissa B. Harrell, Cheryl L. Perry

**Affiliations:** Author Affiliations: Melissa Harrell, Cheryl L. Perry, University of Texas School of Public Health, Austin Regional Campus, Austin, Texas.

## Abstract

**Introduction:**

An understanding of the real-world use of electronic cigarettes (e-cigarettes) is needed to inform surveillance efforts and future state and federal regulation. This study investigates the behavioral aspects of e-cigarette use.

**Methods:**

We used qualitative methods to examine salient characteristics of e-cigarette use. The lead investigator (M.C.) conducted in-depth, semistructured individual interviews to explore patterns and behaviors associated with e-cigarette use among a purposive sample of 50 current adult users. Thematic content analysis was used to analyze qualitative data and document themes.

**Results:**

Several important themes emerged. Although most users started with “closed system” products, the majority switched from that type of e-cigarette to “open system” devices. Responses were diverse on preferred flavors, although mixing flavors was a common practice. Many users had difficulty estimating the total amount of e-liquid they used within a given period and described an iterative process in which they experimented with different nicotine levels to determine their preferred concentration. Reported frequency of use and puffing behaviors varied greatly between users and also differed from the way traditional cigarettes are smoked.

**Conclusion:**

Results from this study have implications for developing appropriate survey metrics for e-cigarette surveillance, the regulation of flavorings, and reporting of e-cigarette product constituents.

## Introduction

Despite the increasing prevalence of electronic cigarette (e-cigarette) use, particularly among young adults (1), little is known about their real-world use (2). For example, information is lacking on preferred product types (“closed-system” or first generation models, which use disposable e-liquid cartridges that require replacement, versus “open-system” or second and third generation models, which require the user to refill the e-cigarette tank). There also are gaps in knowledge of prevalent e-cigarette use patterns and characteristics of various liquid nicotine solutions used. Comprehensive understanding of e-cigarette use is complicated by the numerous options in e-cigarette products (over 460 brands of e-cigarettes with a variety of nicotine levels, cartridge sizes, flavorings, and batteries [3]), as well as anecdotal reporting of modifications to the e-cigarette, such as dripping e-liquids onto the atomizer for enhanced nicotine delivery. Measuring the use patterns associated with these novel devices is challenging. To date, metrics used to quantify e-cigarette use have consisted of such data as puffs per day, periods of use per day, number of liquid cartridges used per day, and milliliters of liquid used per day (4), with little verification of the reliability and validity of such measures.

Research has also begun to explore the characteristics of e-cigarette refill solutions, but several questions remain. Although one study found that e-cigarette users believe that variability in flavors is important for smoking cessation because it increases the appeal of e-cigarettes (5), we have limited understanding of why some people hold this belief. Several studies in controlled settings have assessed the e-liquid nicotine concentration level necessary to mimic nicotine delivery from a conventional cigarette (6–8). However, how findings from these studies pertain to real-world use (eg, common nicotine levels preferred by users) has been explored only minimally.

This study’s aim was to investigate real-world behaviors associated with e-cigarette use in 4 areas: 1) type of e-cigarette products used and modifications made to them; 2) preferred flavors; 3) frequency, quantity and duration of use; and 4) characteristics of e-liquid and nicotine concentrations.

## Methods

### Study design and procedure

In this qualitative study, the lead investigator (M.C.) conducted in-depth, semistructured individual interviews to find patterns and behaviors associated with e-cigarette use among a purposive sample of 50 users. Before the interview, the investigator obtained consent, assured participants that their responses and identity would be kept confidential, and said that there were no right or wrong answers. An independent transcription service transcribed recorded conversations verbatim. Interviews lasted approximately 30 to 45 minutes. The University of Texas institutional review board approved this study.

### Participant eligibility and recruitment

Participants were recruited via a posting on the University of Texas online calendar, a mechanism to advertise research opportunities. Interested individuals were screened online to verify eligibility: being 18 years of age or older and having 6 months of experience with e-cigarettes. Interviews were conducted from December 2014 through April 2015. The lead investigator interviewed 50 people; interviews were terminated as saturation was reached (ie, data collected were determined to be adequate and comprehensive) (9). A $50 gift card was provided as an incentive for participating.

### Interview instrument

The interview instrument was designed to fill gaps in our knowledge of specific aspects of e-cigarette use and to provide information for future studies. As such, predetermined, open-ended questions addressed users’ experiences with e-cigarettes: quantity and duration of use and characteristics of previous and current e-cigarette products such as generation of the e-cigarette device (open system versus closed system), tank or cartridge size, and characteristics of the e-liquid itself (eg, flavorings, nicotine concentration levels).

### Data analysis

QSR NVivo 10 software (QSR International) was used to code transcripts of the interviews for common concepts. Thematic content analysis was used to analyze qualitative data, whereby emerging trends across the data were extracted. The lead investigator conducted all coding. This allowed for a single researcher to be immersed in both data collection and analysis, thereby ensuring that the coding frame adequately described the intentions and content of the interviews (10). First, the lead investigator considered the transcribed data in detail and developed initial codes based on topics present in the semistructured questionnaire. Phrases and sentences were organized into these codes, which allowed the investigator to draw conclusions from the data (11). By using an inductive approach, patterns and relationships were examined within and across codes to generate salient themes.

## Results

Table 1 and Table 2 show demographic characteristics of study participants and patterns of e-cigarette use. Salient themes were type of e-cigarette products and modifications, preferred flavors, ways in which frequency of use and puffing behaviors differ from those for conventional cigarettes, and characteristics of e-liquids and nicotine concentrations**.**

**Table 1:** Demographic Characteristics of Electronic Cigarette (E-Cigarette) Users (N = 50), Study of Real-World Electronic Cigarette Use, United States, 2014–2015

Demographics	n (%)
**Average age (SD)**	28.2 (1.42)
**Sex**	
Male	24 (48%)
Female	26 (52%)
**Race**	
White	31 (62%)
Black	2 (4%)
Asian	9 (18%)
Other	8 (16%)
**Hispanic or Latino**	
No	40 (80%)
Yes	10 (20%)

**Table 2:** Tobacco Use Characteristics of Electronic Cigarette (E-Cigarette) Users, Participants (N = 50) in Study of Real-World Electronic Cigarette Use, United States, 2014–2015

Characteristic	n (%)^a^
**All e-cigarette users**	
**Ever smoked conventional cigarettes?**	
Yes	48 (96%)
No	2 (4%)
**Currently smoke conventional cigarettes?**	
Yes	21 (42%)
No	29 (58%)
**Ever smoked flavored cigarettes?**	
Yes	39 (78%)
No	11 (22%)
**Average months e-cigarette Use, mean (standard deviation)**	16.32 (1.11)
**Intend to quit e-cigarettes?**	
No	33 (66%)
Yes	17 (34%)
**E-cigarette users who also smoke conventional cigarettes (n = 21)**	
**Intend to quit conventional cigarette smoking?**	
No	6 (28%)
Yes	10 (48%)
Don’t know	5 (24%)
**Past 30 days, number of days smoked conventional cigarettes**	
1–5	12 (57%)
6–10	2 (9%)
10–20	1 (5%)
20–30	6 (29%)
**Past 30 days, no. conventional cigarettes smoked per day **	
1–5	16 (76%)
6–10	2 (10%)
>10	3 (14%)
**Former cigarette smokers (n = 27)**	
**How many months since quit smoking cigarettes?**	
<6	4 (15%)
6–12	9 (33%)
>12	12 (45%)
Data missing	2 (7%)

a Unless otherwise indicated.

### Electronic cigarette products and modifications

Nearly all interviewees reported that their first use of e-cigarettes was with a closed-system product. Closed-system e-cigarettes, also referred to as “cigalikes,” are models that require users to dispose of the e-liquid cartridge and replace it with a new, prefilled cartridge. However, few participants reported current use of this type of e-cigarette. The most common type currently used was the open-system device, such as second- or third-generation e-cigarettes, which require the user to refill the e-cigarette tank manually (Table 3). Most respondents in both age groups described the closed-system products as unsatisfying, cheap, or not producing enough vapor. One interviewee (female, age 29) described closed system e-cigarettes as “kind of flimsy, like the ones that Marlboro keeps giving away for free at all the bars.” Another interviewee (female, age 29) noted, “I started, like a lot of people did, with whatever they were selling at Walgreens at the time. And the amount of vapor that you get with that is not satisfying.” Most participants (n = 42) used open system products, such as those with variable voltage batteries, refillable tanks (some of which allow the user to modify the airflow), and more customizable features. As one user (male, age 49) explained, “Well, you can regulate your hits. You can get a bigger hit off of [open system e-cigarettes]. I noticed with the [closed system] it wasn't quite as much smoke or vapor, and I wanted more.”

**Table 3:** First Type of Electronic Cigarette (E-Cigarette) Used and Type Currently Used (N = 50), Study of Real-World Electronic Cigarette Use, United States, 2014–2015

Type	First Product	Current Product
Closed-system e-cigarettes^a^	34 (68%)	8 (16%)
Open-system e-cigarettes^b^	16 (32%)	42 (84%)

a Closed-system e-cigarettes, also referred to as “cigalikes,” require users to dispose of the e-liquid cartridge and replace it with a new, prefilled e-liquid cartridge.

b Open-system e-cigarettes are second- or third-generation models that require the user to refill the e-cigarette tank with e-liquid manually. Open system models are more customizable and allow users to interchange different batteries (some of which have variable voltages) and tanks.

Six participants who used open-system devices described the process of wrapping the heating coil themselves with Kanthal wire, placing organic cotton around the coil, and dripping e-liquids directly onto the cotton before puffing. Four open-system users described modifying their e-cigarettes to enhance airflow. One user (male, age 32) said, “Some devices have smaller air holes, so they will drill them out. If you go into some of the local vape shops, they have a drill machine just sitting there behind the counter. It's that common. Basically, you need surface area and air to generate the clouds. So it's all about trying to get air in and air out.” Many users (n = 24) of open system e-cigarettes had batteries in which voltages could be modified, although when queried about voltage settings, 17 (about 70%) of this group, did not express a desire to use high voltage settings because it yielded a burnt taste or other unpleasant effects. As one user (female, age 29) noted, “When I go to higher voltages, I might get more vape, but at a certain point, it becomes hot, and you'll burn it out really fast. Like cigarettes, there'll be a heat to it. First, I think I was looking for a similar sensation, and now I don't like that.”

### Preferred flavors

Interviewees reported trying a multitude of e-cigarette flavors, such as tobacco flavoring and apple or tea flavorings. Responses were mixed on users’ preference for tobacco flavors versus other flavors. For example, a former smoker (female, age 20), said, “It's like it would tempt me more if I had something that was similar to a cigarette, because I'm trying to get away from that.” Others emphasized the importance of an e-cigarette tasting like a traditional cigarette, as another former smoker (female, age 36) said, “It was an easier conversion just to go with tobacco flavoring.”

Many participants (n = 22) used mixtures of different flavors together, mixed either by an employee at a local vape store or by buying separate off-the-shelf e-liquid solutions to mix. One participant described making his own e-liquid from separately bought components (pharmaceutical-grade liquid nicotine, vegetable glycerin, and food-grade flavoring). Others described adding other constituents such as vanilla, vodka, distilled water, or liquid caffeine drops to their e-liquid to achieve different effects.

Five interviewees described undesirable experiences with e-cigarette flavoring, which ranged from generally unpleasant effects such as nausea after using strawberries and cream (male, age 29, and female, age 20) and strawberries and honey (male, age 20) to more serious effects such as a burning sensation (female, age 23) and throat irritation (female, age 21) after using a cinnamon-flavored e-cigarette. Two interviewees described the flavor experience as playing an instrumental role in cigarette smoking cessation. As one user (female, age 21) described, “If I don't like the flavor, I'm going to smoke a cigarette in a weird way, because it's not satisfying. It's like I'm a slave to nicotine, but if you find a flavor that you like, you're more inclined to be like, ‘This is sufficient. I don't want [a cigarette].’”

### Frequency of use and puffing behaviors differ from conventional cigarettes

Participants were asked to describe how frequently they used e-cigarettes in terms of times per day, length of each session, and approximate number of puffs. Reported patterns of use varied greatly among users but were typically distinctive from the way traditional cigarettes are smoked. For example, one user (female, age 29) described using the e-cigarette more frequently than a conventional cigarette: “It's just kind of always there. I almost do it without thinking about it now. I don't go pick it up, and intentionally vape for five or ten minutes, set it down, and go do something else.” Another user (male, age 49) described short but frequent sessions of use: “I probably have about 2 or 3 puffs and that's it, once every 30 to 45 minutes throughout the day.” In fact, this pattern of taking only a few puffs was described by at least 6 other users. For example, one female (age 25) noted “It creates the same sensation that a cigarette does, but in just 1 or 2 puffs as opposed to the whole cigarette.” In addition, the type of e-cigarette device used affected puffing behaviors, as one user (female, age 23) explained: “Even though I was using the same strength of nicotine juice, with this [open-system e-cigarette], it was more powerful, and rather than having to take several puffs, I would only have to take one to really feel the effect.”

### Characteristics of e-liquid and nicotine concentrations

Across types of e-cigarette products and user age groups, users had difficulty estimating the amount of e-liquid they used within a given period. People who used closed-system products frequently did not know how much e-liquid each cartridge contained, so they gave their best estimate of the duration of the cartridge. Fourteen participants who used open-system e-cigarettes recalled how long one bottle of e-liquid lasted rather than how much they consumed in one day. For example, one 19-year-old man said, “I don't know the exact volume. I get the bottles which are —I think they're ten milliliters, I don't know. It's the normal size, the smallest normal size. And I probably go through a sixth of it every day.” Sixteen users with open-system e-cigarettes did not know how much e-liquid their tank held. As one user (male, age 49) illustrates, “I don't know how much is in a tank, but I'm sure that I go through about one-half to three-quarters of a tank a day.”

Estimating quantity of use was complicated by other factors such as sharing e-cigarette devices or e-liquids with roommates, friends, or significant others. In addition, 3 users reported that they used different types of e-cigarette devices concurrently. Moreover, one user (male, age 28) described the composition of the e-liquid itself as a factor in how long the e-liquid lasted: “If it's high in [vegetable glycerin], it's thicker and it burns much faster, so you can go through it quickly.”

Interviewees were asked to describe their current or most common concentration of nicotine used as well as patterns of nicotine levels they had tried over time. Some users (n = 9), particularly those who used closed-system products, reported that they did not know the concentration of nicotine in their e-cigarette. Those who bought the e-liquid separately for an open-system e-cigarette were more frequently able to report knowing the level of nicotine they used because it was listed on the bottle or because they requested a specific nicotine concentration from their local vape shop.

 Among the 17 participants with intentions to eventually quit using the e-cigarette, 13 described scaling down their nicotine level. One participant (male, age 49) said “Maybe eventually I'll even quit e-cigarettes. So after the first 6 months, instead of a 32, I stepped down to 24, and then after another 3 months, I stepped down to 18.” Many users (n = 18) described an iterative process in which they experimented with different nicotine levels, as one (male, age 28) described, “‘Okay. Twelve, I'm still feeling a bit sick on this, so I'll drop it to 9, and that's about right.’ And then I was like, ‘Well, I'm going to take a risk and go with 3. No, I’m not ready yet; let me go to 6.’ Just kind of experiment until you get down to where you want to be.” Frequently, users reported that high nicotine concentrations caused uncomfortable effects such as a harsh sensation in the throat, headaches, or dizziness. However, this threshold differed among users.

## Discussion

Interview findings paint a picture of real-world e-cigarette use among adults. The themes identified can help us understand what features, products, and flavors e-cigarette users find desirable. Findings show that even experienced users have difficulty estimating the volume of their e-liquid intake and that determining the concentration of nicotine in their e-liquid is often the result of experimentation with different levels. Results from this study have implications for measuring e-cigarette use and future regulation.

Participants in this study were almost unanimous in their preference for open-system e-cigarettes over closed-system products. These findings align with a recent survey of more than 4,000 adult e-cigarette users that concluded that most users transitioned to more advanced devices primarily for the purpose of obtaining a “more satisfying hit” (12). However, current US tobacco surveys, such as the National Adult Tobacco Survey (13) and *HealthStyles* (14)*,* do not collect information on the type of e-cigarette used. Given the rapidly changing technology of e-cigarettes, trends in e-cigarette use should be re-evaluated frequently, and surveillance measures should include the most recent and popular products on the market.

This study’s findings add to the limited literature suggesting that puffing behaviors with e-cigarettes differ from those with conventional cigarettes (2), which will help to develop metrics tailored for e-cigarettes. Although this study did not use a topography instrument, user reports alone suggest that one pattern of e-cigarette use, as described by 7 participants or 14% of our sample, is frequent instances of only a few puffs. This is consistent with research that found that 17% of e-cigarette users sampled reported using e-cigarettes more frequently than conventional cigarettes but taking fewer puffs on each occasion (15). It was previously documented that e-cigarette product characteristics, such as device voltage and resistance and a large range of nicotine concentrations, affect nicotine yield (8). Therefore, it is possible that users in this study may have optimized such product characteristics to achieve desired subjective effects with only a few puffs from the e-cigarette. In terms of surveillance, these findings demonstrate that although users may report many instances of use of e-cigarettes per day, each instance may actually be brief.

Findings from these interviews also revealed that those who used open-system products had great difficulty in quantifying how much e-liquid they used in a given period and were more likely to report how many days or weeks a bottle of e-liquid lasted (although many could not recall the exact volume of the bottle) rather than how much e-liquid they consumed in one day. Users also reported highly variable periods of use per day, which is consistent with a pilot study on real-world e-cigarette use behaviors (16). Considering these findings, it may be more appropriate to focus on measures of intensity of e-cigarette use rather than on estimated milliliters of daily, weekly, or monthly e-liquid intake or number of puffs. Such measures assess how intensively and for how long an individual has used an e-cigarette, differentiating between intensive (daily for at least 1 month), intermittent (more than once or twice but not daily for a month or more), and non-use or at most, once or twice (17). Furthermore, as study findings showed, daily patterns of e-cigarette use vary greatly, so additional measurement of intensity of daily use is necessary to accurately understand differences among users. A future metric could be near constant daily use (ie, 20 or more times per day), heavy daily use (ie, 10–19 times per day), occasional daily use (ie, 5–9 times per day), and sparse daily use (ie, 1–4 times per day). Future research is needed to explore the reliability and validity of such intensity measures.

The US Food and Drug Administration proposed extending regulatory authority, established by the Family Smoking Prevention Tobacco Control Act of 2009, to other tobacco products, including e-cigarettes (18). Findings from this study offer several considerations for e-cigarette regulation. A large number of participants reported enjoying e-cigarette flavors, including some who described flavor as an important part of quiting smoking conventional cigarettes. These findings are consistent with a focus group study of young adults that found that favorable perceptions of e-cigarettes were due in part to the availability of flavoring (19). Federal regulators must weigh these findings both in future consideration of restricting e-cigarette flavorings (a desirable feature in a potentially harmful product) and efforts to better understand the role of flavoring in smoking cessation and harm reduction. Regulatory restriction of e-cigarette flavorings should consider the range of consequences of such policy actions, which may be positive for some populations (ie, those trying to quit conventional cigarettes) but negative for others (ie, young people who may not otherwise try tobacco and nicotine products).

Regulating e-cigarettes, including reporting the constituents in these products, is a crucial next step to assessing their potential harm. Research shows that constituents vary across e-cigarette products (20), and when discussing their choices among various nicotine and flavoring options, users in this sample reported mild adverse effects, ranging from headaches caused by high nicotine concentrations to a burning or nauseating sensation resulting from certain flavorings. A clear understanding of the constituents and their respective levels is necessary for evaluating the potential harm of e-cigarettes. Finally, labeling the contents of e-cigarettes, including the concentration level of nicotine, would warn people of the potential deleterious effects of e-cigarettes.

Because of its qualitative methodology, our study results are not generalizable outside the small study population. Furthermore, data were self-reported. Future research should, use more robust designs to further explore the salient themes from population-based samples, include users who are under age 18, and have more than one coder assess the reliability of the coding process. Nevertheless, the themes discussed expand understanding of e-cigarette use. Our findings highlight the need to ensure that e-cigarette surveillance and regulation reflect the way these products are used in real-world circumstances. Specifically, results should be considered when developing appropriate survey metrics, which are particularly important for keeping pace with new products, regulating flavorings, and reporting product constituents.
